# Innovative 3D-Printed Superhydrophobic Porous Architectures for Continuous Oil–Water Separation

**DOI:** 10.3390/polym17111465

**Published:** 2025-05-25

**Authors:** Xiaolong Wang, Jingjing An, Alaa Hassan, Qingsen Gao, Xianhu Liu, Hakim Boudaoud

**Affiliations:** 1 Équipe de Recherche sur les Processus Innovatifs (ERPI), Université de Lorraine, F-54000 Nancy, France; xiaolong.wang@univ-lorraine.fr (X.W.); alaa.hassan@univ-lorraine.fr (A.H.); hakim.boudaoud@univ-lorraine.fr (H.B.); 2 College of Materials Science and Engineering, Zhengzhou University, Zhengzhou 450001, China; 15138543202@163.com; 3 Institute of Polymer Materials, Friedrich-Alexander-University Erlangen-Nuremberg, Martensstr. 7, 91058 Erlangen, Germany; qingsen.gao@fau.de

**Keywords:** 3D printing, superhydrophobicity, oil–water separation, fused filament fabrication, additive manufacturing

## Abstract

Efficient superhydrophobic oil–water separation materials are essential for environmental remediation and industrial wastewater treatment. In this study, by optimizing printing parameters, such as printing speed, extrusion multiplier, and layer height, we achieved high-precision 3D porous architectures with uniform pore sizes. The pore size could reach 677.3 µm, with a maximum deviation of less than 36.1 µm. Additionally, we successfully printed pores as small as 186.7 µm, representing the smallest FFF-printed pore size reported in the literature. The printed structures were modified using a spray-coating method, achieving a superhydrophobic surface with a water contact angle of 158.2°. The material was tested in a continuous oil–water separation system, maintaining stable oil removal performance for 24 h. The highest separation efficiency reached 88.6%, demonstrating strong durability and long-term applicability. This study establishes a scalable, low-cost approach for fabricating highly efficient 3D superhydrophobic porous materials, offering new opportunities for continuous oil spill cleanup and industrial wastewater treatment.

## 1. Introduction

Oil–water separation has become a critical environmental and industrial challenge due to the increasing discharge of oily wastewater and frequent oil spill accidents. These issues pose severe threats to aquatic ecosystems and human health, necessitating the development of efficient and sustainable separation technologies [1]. Traditional oil–water separation methods, such as coagulation [2], flotation [3], and in situ burning [4], often suffer from high energy consumption, low separation efficiency, and complex operational processes.

Inspired by natural surfaces such as lotus leaves and water-strider legs, superhydrophobic materials with a water contact angle (CA) higher than 150°, such as aerogels [5], foams [6,7], microspheres [8], and membranes [9,10], have demonstrated exceptional potential in the oil–water separation field due to their ability to selectively repel water while permitting oil to pass through. In these works, membranes enable rapid filtration-based oil–water separation, provide structural flexibility, and offer reusability [11]. However, in practical applications, most existing studies have focused on the fabrication of superhydrophobic 2D structure, which faces significant limitations in achieving continuous oil–water separation. These materials typically require specialized apparatus for operation and consume considerable energy to collect and filter water, making the separation process less efficient and resource-intensive. More importantly, their applicability is constrained, as they cannot be directly deployed in contaminated water bodies, limiting their effectiveness in large-scale environmental remediation. Therefore, the fabrication of architecturally structured superhydrophobic mesh-based oil collection devices presents an effective solution to this challenge. We propose that by transforming the conventional 2D superhydrophobic mesh into a three-dimensional (3D) structured design, such as a cubic framework, it can be directly deployed as a contaminant collector in polluted water bodies, such as rivers, for continuous oil cleaning. To fabricate such porous architecture, 3D printing emerges as an ideal candidate due to its ability to customize and control structural geometry [12,13]. This technology has been widely applied in various fields, including biomedical engineering [14], aerospace [15], and environmental science [16], owing to its rapid prototyping capabilities and design flexibility. However, most previous studies have predominantly focused on 2D superhydrophobic membranes, failing to fully exploit the advantages of 3D printing for constructing architecturally designed porous structures [17,18,19]. Only a limited number of studies have reported oil–water separation devices with 3D-structured architectures. Li and co-workers demonstrate an interesting 3D printing device for oil–water separation [20]. However, the 3D filter is just an idea. Xu et al. reported a smart device consisting of a 3D printing polyvinylidene difluoride (PVDF) skeleton and a hydrophobic coated copper mesh [21]. In this work, the 3D porous component is composed of modified hydrophobic copper mesh, rather than being 3D printed. Inspired by the water collecting behavior of cactus, Shin and co-workers fabricated a hollow polydimethylsiloxane (PDMS) sponge with a hydrophobic surface, which has more space for the storage of collected oil [22]. In the preparation process, 3D printing is only used to create a mold for PDMS. As discussed above, the development of 3D-printed 3D porous structures for oil–water separation has not yet been reported. Printing 3D porous structures still presents certain challenges.

In this study, we present an innovative 3D-printed porous architecture for continuous oil–water separation. Although various high-precision 3D printing technologies, such as stereolithography (SLA) [23], selective laser sintering (SLS) [24], and digital light processing (DLP) [25], offer excellent printing accuracy, we opted for Fused Filament Fabrication (FFF) due to its low cost, ease of use, and suitability for large-scale industrial production [26]. These advantages make FFF highly promising for practical applications in oil–water separation. Polylactic acid (PLA) was chosen as the base polymer due to its biocompatibility, biodegradability, and favorable melt rheology for FFF printing. Its thermal properties (Tg ≈ 60 °C, Tm ≈ 160–170 °C) and moderate viscosity under extrusion conditions enable good dimensional control during pore formation. To ensure effective oil–water separation, the porous structures must have small pore sizes to achieve high intrusion pressures [27]. However, the relatively lower resolution of FFF presents challenges in fabricating precise microstructures with uniform pores [28]. To overcome these limitations, we employed precise computational modeling and optimized printing parameters, including printing speed, printing temperature, and extrusion multiplier, to fabricate cubic porous structures with uniform square pores and minimal defects. The fabricated structures exhibited pore heights ranging from 543.2 µm to a maximum of 677.3 µm. Furthermore, by fine-tuning the printing parameters and model design, we successfully produced samples with pore heights as small as 186.7 µm. Additionally, to enhance printing efficiency, we optimized the layer height during fabrication, achieving a significant 83.60% reduction in printing time while maintaining a minimum pore height of 287.7 µm. This study represents the first report of directly fabricating 3D porous structures with uniform pores (error < 36.1 µm) using FFF technology, achieving smaller pore sizes than previously reported studies. Furthermore, to impart superhydrophobicity to the fabricated structures, we successfully modified surfaces using a spray-coating method with nano-silica (SiO_2_) particles, achieving a remarkable water contact angle of 158.2°. The mechanical stability of the coated layer was validated through peeling tests, confirming its durability under operational conditions. The resulting superhydrophobic porous structures demonstrated excellent oil–water separation performance across various applications. Notably, in a continuous oil–water separation system driven by suction pumping, the material maintained stable performance for at least 24 h, achieving a separation efficiency of 87.2%.

This study represents the first report of directly fabricating 3D porous architectures with uniform pores using FFF technology, achieving smaller pore sizes and higher structural precision than previously reported studies. The findings provide a new pathway for scalable, low-cost, and efficient superhydrophobic oil–water separation materials, offering potential applications in environmental remediation and industrial wastewater treatment.

## 2. Materials and Methods

### 2.1. Material

The filament used is PLA (Raise Premium 1.75 mm PLA filament, Raise3D, Irvine, CA, USA). PDMS and the curing agent were purchased from Dow Corning Co., Ltd., Shanghai, China. Silica nanoparticles (Si-300NM) were purchased from Lanling Chemical Co., Ltd., Foshan, China. Ethanol, Isopropanol, and other solvents were purchased from Zhiyuan Reagent Co., Ltd., Tianjin, China. Commercial sponges were purchased from Aojiawei Co., Ltd., Shanghai, China. All materials were used as received.

### 2.2. Fabrication of 3D PLA Porous Architectures

**Figure 1:**
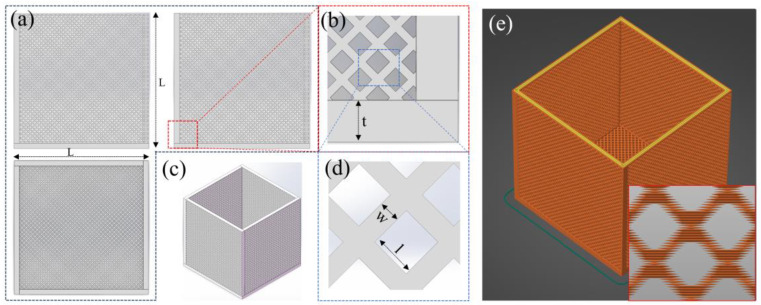
(**a**) Three-view schematic diagram of the diamond pore structure; (**b**) detailed top-view illustration; (**c**) complete 3D model of the sample; (**d**) magnified detail of the pore structure; (**e**) digital model of the sliced sample prepared for 3D printing.

The 3D printing experiments were conducted using a commercial dual-extruder FFF printer (Raise 3D E2). As illustrated in Figure 1a–e and Figure S1a–e, hollow cube-shaped models featuring diamond and square pore structures were designed with SolidWorks 2021 software. In the schematic, ‘L’ denotes the edge length of the cube, ‘t’ represents the thickness of the porous vertical walls, ‘w’ indicates the width of the solid portion within the porous structure, and ‘l’ stands for the edge length of the pore openings. To precisely investigate the variations in pore structures under different printing conditions, various printing parameters were designed, as summarized in Table 1 (diamond pores) and Table S1 (square pores).

### 2.3. Hydrophobic Modification

In the modification process, silica nanoparticles (0.3 g) and isopropanol (10 mL) were initially dispersed into ethanol (10 mL), followed by ultrasonication for 15 min. Subsequently, PDMS (0.07 g) and curing agent (0.007 g) were added, and the mixture was ultrasonicated for another 30 min to achieve a uniform dispersion. For spray coating, this dispersion was transferred into a commercial spray gun and sprayed onto the sample from approximately 20 cm above its surface at a pressure of 0.2 MPa. The spray gun was slowly moved across the substrate surface for a total of 20 cycles. Finally, the coated sample was dried in a fume hood for more than 8 h to ensure the complete evaporation of ethanol.

### 2.4. Characterizations

The contact angle measurements were conducted at room temperature using a goniometer (SL200KS, KINO Industry Co., Ltd., Norcross, GA, USA), employing droplets of 2.5 μL volume. The morphological analysis of the samples was performed via scanning electron microscopy (SEM, Hitachi S-4800, Hitachi High-Technologies Corporation, Tokyo, Japan), operated at an accelerating voltage of 20 kV. Additionally, surface morphology and elemental distribution were characterized through SEM and energy-dispersive X-ray spectroscopy (EDS) mapping (SEM, Quanta 400 FEG, FEI Company, Hillsboro, OR, USA). Pore sizes were analyzed quantitatively using Nano Measurer software 1.2 (Fudan University, Shanghai, China) based on SEM images.

### 2.5. Oil–Water Separation Test

In the pumping oil–water separation experiment, the sample (D3LTE) was placed in a mixture of 400 mL of water (dyed with methylene blue) and 40 mL of cyclohexane (dyed with Sudan III). The oil separated from the sample was continuously collected using an electric pump (KCP-B08 from Karmoel Co., Ltd., Shanghai, China). The separation flow rate (*f*) was calculated according to the following equation:

$$f=\frac{V}{t}$$

where *V* represents the volume of separated organic solvent, and *t* is the separation time, defined as the interval from the beginning of the experiment until no further cyclohexane could be collected by the electric pump.

The separation efficiency (*e*) was calculated using the following equation:

$$e=\frac{m}{M}$$

where *M* and *m* represent the weight of cyclohexane before and after the separation process, respectively.

In the continuous pumping oil–water separation test, the sample was placed in 440 mL cyclohexane, and the cyclohexane was continuously collected by an electric pump and recirculated back into the beaker, allowing continuous flow across the sample surface. At different time intervals, the sample was taken out and subjected to the standard pumping oil–water separation test mentioned above to evaluate its separation efficiency over time.

## 3. Results and Discussion

### 3.1. Fabrication of Porous Architectures

First, we should analyze the challenges associated with printing vertical porous structures using FFF technology. FFF technology involves the deposition of molten polymer layer by layer, as controlled by a computer program, to form the final structure. This technique typically requires support structures when printing overhangs [29]. However, vertical porous structures inevitably contain numerous overhangs. Printing these unsupported overhangs can lead to material deformation, while using supports would make it impossible to achieve the desired porous structures. Additionally, the precision of FFF is relatively low, as its motors cannot accurately control the extrusion output rate of the polymer. To address these issues, we propose two solutions. First, we could try to precisely adjust the relevant printing parameters, such as printing speed, printing temperature, and extrusion multiplier. Second, we can modify the pore shapes in the design to minimize overhangs.

In this work, as shown in Figure 1a–e and Figure S1a–e, we primarily designed two different pore shape models. One model has square pores, which are simpler to model, but as indicated by the red box in Figure S2a, it includes some overhang structures. The other model has diamond-shaped pores, as shown in Figure S2b. Although more challenging to model, its 45° inclined structure is not considered an overhang in FFF technology.

The porous architecture of oil–water separation materials plays a pivotal role, as an optimal pore design should selectively hinder water infiltration while allowing oil to pass through. As discussed in our previous work on invasion pressure theory [30], larger pores generate weaker negative capillary forces, whereas smaller pores exhibit stronger capillary resistance. Consequently, different pore structures correspond to varying capacities for withstanding water pressure. Meanwhile, the pore size also influences the flow rate during oil–water separation, which will be further discussed in Section 3.3: Oil–water separation experiment. In FFF technology, the minimum pore size is typically set to twice the nozzle diameter. In this study, a 0.4 mm diameter nozzle was used. Although a 0.2 mm nozzle provides higher precision, it is less commonly employed. Therefore, unless otherwise specified, l in Figure 1 and Figure S1 is set to 0.8 mm. To enhance flux while maintaining hydrophobicity, the number of pores should be maximized. Accordingly, w is set to 0.4 mm, corresponding to the 0.4 mm nozzle diameter.

In the first set of experiments, samples of square pore structures under various parameters, as detailed in Table S1, were printed. Among these parameters, the rationale for the setting of t (the thickness of the wall) will be discussed in the diamond pore structure section. Printing speed, printing temperature, and extrusion multiplier are all set to the default recommended settings of the printer. To enhance model accuracy and facilitate observation, the layer height was set to a minimum of 0.05 mm, despite the longer printing time required. As shown in Figure S1f, S1 failed to form the intended pore structure, with horizontal layers completely covering the pores. This issue is attributed to the limited precision of FFF technology, where excessive filament extrusion occurs during printing. To improve print quality, adjustments to printing speed, temperature, and extrusion multiplier can be made. Reducing the printing speed enhances print quality [31]. However, when printing microstructures, a speed below 30 mm/s leads to significant deterioration due to the insufficient precision of FFF motors, which struggle to extrude small amounts of polymer accurately at lower speeds. The layer covering of pores may also result from polymer stringing, which can be mitigated by lowering the printing temperature. The minimum printing temperature for PLA is 205 °C, as demonstrated in S3 of Figure S1h, where reducing the temperature alleviates pore coverage. Adjusting the extrusion multiplier further controls polymer extrusion. When set to 0.9—the lowest practical limit—pore coverage is reduced, as shown in S2 of Figure S1g, though significant polymer stringing remains. A combination of lowering both the temperature and extrusion multiplier (S4 in Figure S1i) optimizes both pore coverage and stringing, yet notable defects and overhang issues persist. At this stage, all adjustable parameters have been explored, leading to the conclusion that the square pore structure is unsuitable for vertical porous designs.

**Figure 2:**
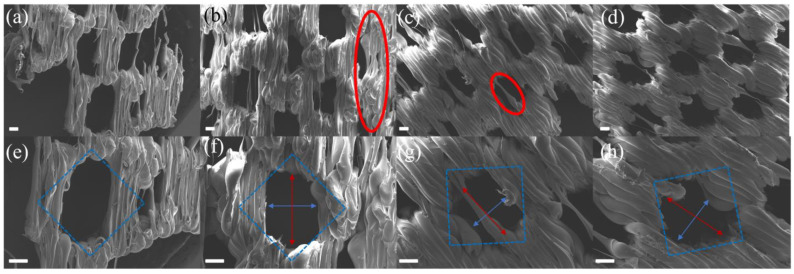
SEM images of diamond pore structure. (**a**,**e**) D1, (**b**,**f**) D2, (**c**,**g**) D3, and (**d**,**h**) D4. Scale bar is 200 µm. (Blue box: designed pore size in the model; red circle: defect; blue arrow: pore height; red arrow: pore width, respectively).

Table 1 presents the parameters used for printing the diamond pore structures. D1, D2, D3, and D4 were printed under the same conditions but with different model thicknesses (t in Figure 1). As shown in Figure 2a,e, D1, with a thickness of 0.8 mm, was not successfully printed, and many pore structures were missing. This failure is due to the principles of FFF technology; when the thickness of the previous layer is too low, its mechanical properties are insufficient to support the deposition of the next layer, leading to print failure. As illustrated in Figure 2b,f, when the thickness was 1.6 mm, the print quality of D2 was low, with numerous defects still present. With thicknesses of 2 mm and 2.4 mm, shown in Figure 2c,d,g,h, the material was successfully printed, but defects were still observed in the pore structures. Therefore, in subsequent experiments, models with a thickness of 2 mm will be used to balance printing success and time efficiency.

**Figure 3:**
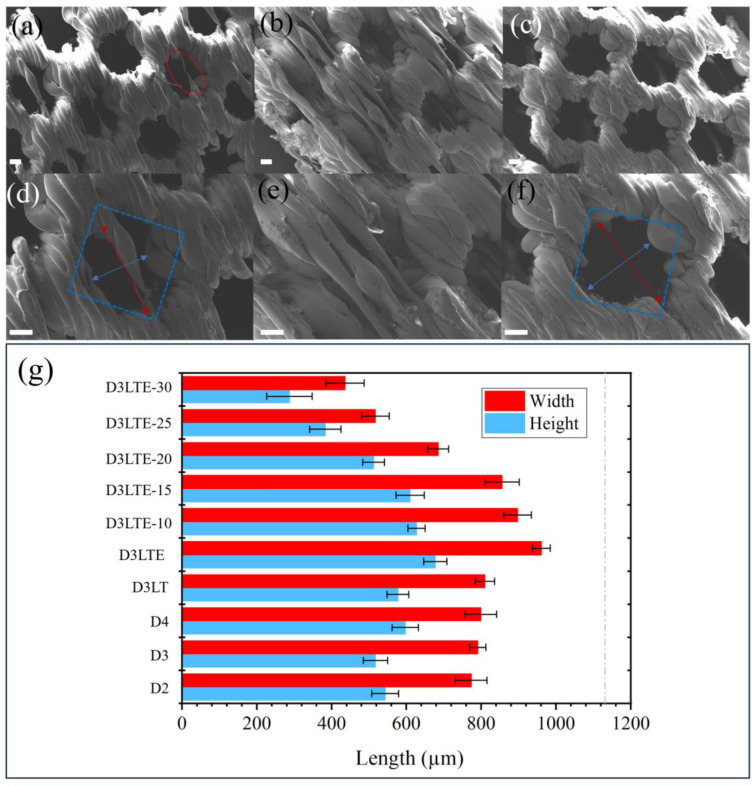
SEM images of diamond pore structure. (**a**,**d**) D3LT, (**b**,**e**) D3LTF, and (**c**,**f**) D3LTE. Scale bar is 200 µm. (Blue box: designed pore size in the model; red circle: defect; blue arrow: pore height; red arrow: pore width, respectively). (**g**) Pore size distribution graph of different samples.

In the subsequent experiments, based on the printing parameters of D3, the printing temperature was reduced to 205 °C to fabricate D3LT. As shown in Figure 3a,d, D3LT was successfully printed without a significant decline in quality compared to D3. To enhance energy efficiency, further optimizations will be based on D3LT. To improve printing efficiency, D3LTF was printed at a higher speed than D3LT. However, as clearly illustrated in Figure 3b,e, this led to a substantial deterioration in print quality, confirming that a speed of 30 mm/s is optimal. Additionally, the extrusion multiplier was adjusted to fabricate D3LTE, which significantly improved print quality, as evidenced in Figure 3c,f.

As shown in Figure 3g, the measured pore widths of D2, D3, D4, D3LT, and D3LTE are 773.2 µm, 790.6 µm, 798.9 µm, 810.2 µm, and 961.2 µm, respectively, while the corresponding pore heights are 543.2 µm, 516.9 µm, 596.9 µm, 577.4 µm, and 677.3 µm. SEM images in Figure 2 and Figure 3 indicate that the actual pore sizes are smaller than the designed values due to the limited precision of FFF technology and polymer deformation. However, reducing the extrusion multiplier effectively increases pore sizes, bringing them closer to the intended dimensions. Compared to square pores, diamond-shaped pores exhibit superior print quality.

As illustrated in Figure S2, L1 and L2 represent segments printed in a single print job. Diamond pores demonstrate a 41.4% larger volume per print job compared to square pores. Due to the resolution limitations of FFF technology, printing square pore structures requires the nozzle to extrude smaller amounts of polymer per segment, which can lead to reduced print quality. The diamond structure mitigates this issue, improving overall print fidelity. Additionally, the absence of overhangs in diamond pore structures further enhances print quality.

**Figure 4:**
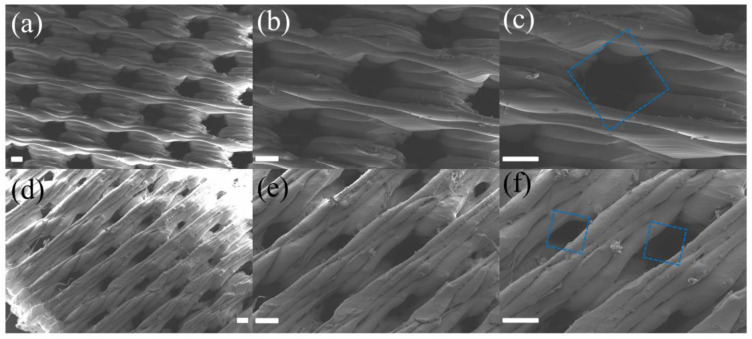
SEM images of diamond pore structure with (**a**–**c**) 0.4 mm and (**d**–**f**) 0.2 mm pore height. Scale bar is 200 µm. (Blue box: designed pore size in the model.)

Subsequently, we conducted preliminary printing experiments with smaller pore sizes, based on the parameters of D3LTE, while only varying the pore size. Samples with pore sizes of 0.4 mm and 0.2 mm were successfully printed. As shown in Figure 4, the porous structures exhibited high print quality. The samples with a designed pore size of 400 µm exhibited uniformly printed pores with an average diameter of 186.7 µm. However, when the pore size was reduced to 0.2 mm, the pore sizes became inconsistent, and many pores were not fully through-holes. Although a vertical porous structure with a pore size of 186.73 µm was successfully fabricated through the precise control of printing parameters, its inability to significantly reduce printing time by adjusting the layer height presents potential challenges in practical oil–water separation applications. Due to limitations such as reduced flow rate and increased fabrication complexity observed in smaller pore sizes, subsequent studies will primarily focus on samples with a pore size of 800 µm, which offer a more favorable balance between performance and manufacturability.

**Figure 5:**
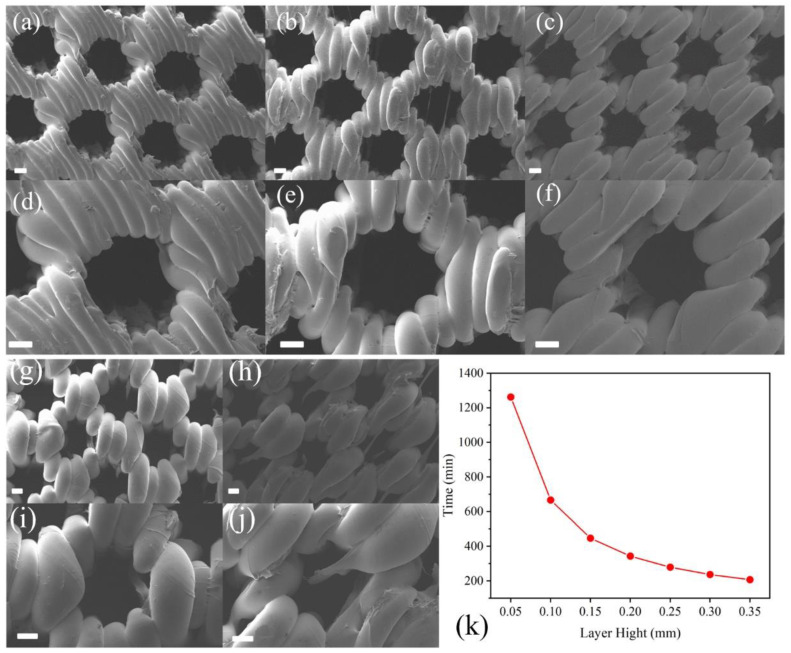
SEM images of diamond pore structure. (**a**,**d**) D3LTE-10, (**b**,**e**) D3LTE-15, (**c**,**f**) D3LTE-20, (**g**,**i**) D3LTE-25, and (**h**,**j**) D3LTE-30. Scale bar is 200 µm. (**k**) Relationship between layer height and printing time.

Reducing the fabrication time of vertical porous structures is a critical step toward achieving large-scale industrial production, significantly enhancing manufacturing efficiency. Among printing parameters, increasing the printing speed is a direct approach to reducing fabrication time. However, as shown in Figure 3b,e, when the speed exceeds 30 mm/s, substantial structural defects emerge due to the precision limitations of FFF technology. An alternative method currently being considered for time reduction is decreasing the layer height. As presented in Table 1, samples such as D3LTE-10 were designed based on the printing parameters of D3LTE, with D3LTE-30 featuring a layer height of 0.3 mm—the maximum limit supported by the employed printer. As illustrated in Figure 5k, adjusting the layer height effectively reduces printing time. The printing time for D3LTE with a layer height of 0.05 mm is 1262 min, whereas for D3LTE-30 with a 0.3 mm layer height, it is reduced to 207 min—representing an 83.60% reduction in printing time. This significant time reduction is attributed to the layer-by-layer deposition mechanism of FFF technology, where a larger layer height results in fewer total layers, thereby shortening the overall printing duration. However, increasing the layer height also compromises print resolution. The SEM images in Figure 5a–j depict samples printed with different layer heights, demonstrating that all exhibit well-formed and structurally intact pores due to optimized printing parameters. Nonetheless, as shown in Figure 3g, D3LTE-10 has a pore height of 627.4 µm, whereas D3LTE-30 exhibits a significantly smaller pore height of 287.7 µm. The increased layer height introduces larger dimensional deviations, leading to a notable reduction in the actual pore size compared to the designed model. Moreover, as observed in Figure 5g–j, an increased occurrence of filament stringing and other printing defects can be identified. These imperfections are also attributed to the precision limitations of FFF technology during the printing process.

In addition to performance optimization, the cost-effectiveness of this fabrication method is also noteworthy. The FFF printer used in this study is commercially available at a market price ranging from approximately $165 to $275 USD, and the PLA filament costs around $38 USD per kilogram, resulting in a material cost of approximately $0.68 USD per sample. These figures highlight the economic viability of the method, making it highly attractive for large-scale production and field applications in environmental remediation and industrial wastewater treatment.

### 3.2. Hydrophobicity

**Figure 6:**
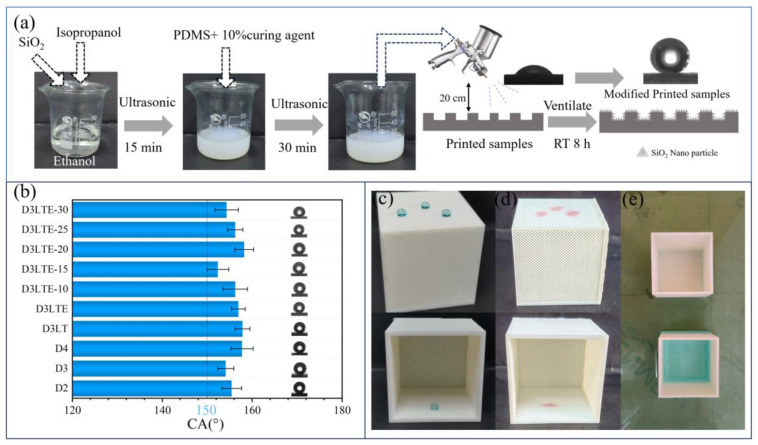
(**a**) Hydrophobic modification process and comparison of water contact angles before and after modification of D3LTE. (**b**) Water contact angle of different samples. Digital photograph of D3LTE and (**c**) its water contact angle and (**d**) oil contact angle (cyclohexane). (**e**) Photographs of the D3LTE surface submerged in water before and after modification.

In our previous work, the spray-coating method has been demonstrated as an efficient and cost-effective approach for superhydrophobic modification [32], as illustrated in Figure 6a. Ethanol, an environmentally friendly and low-cost solvent, was selected as the dispersion medium. The addition of isopropanol enhances the dispersion of silica nanoparticles in ethanol, improving coating uniformity. PDMS acts as a binder, anchoring the silica nanoparticles to the sample surface. Silica nanoparticles contribute to reducing the surface energy of the material while simultaneously increasing nanoscale roughness. According to the Cassie and Wenzel models [33], this dual effect significantly enhances the hydrophobicity of the modified surface. However, the long-term stability of the silica nanoparticles on the surface, particularly their potential detachment during practical applications, remains a concern. Further investigations are required to assess the extent of nanoparticle loss and its potential environmental impact.

As shown in Figure 6a, after hydrophobic modification, the contact angle of D3LTE increased from 79.2° to 156.9°, reaching the threshold for superhydrophobicity. The digital photograph in Figure 6c illustrates that water droplets retain a spherical shape on the modified D3LTE surface, indicating a strong water-repellent effect. For oils such as cyclohexane, as depicted in Figure 6d, the liquid is completely absorbed due to capillary forces, resulting in a contact angle of 0°. Furthermore, Figure 6e presents a comparison of D3LTE before and after modification when submerged in water. The unmodified sample exhibits water infiltration into its internal pores, whereas the modified D3LTE remains dry. This is attributed to the negative capillary effect generated by its superhydrophobic surface, which effectively resists water penetration under hydrostatic pressure.

Except for D3LTF, where the pore structure was heavily obstructed, and D1, which failed to print successfully, the contact angles of all other samples were measured. As shown in Figure 6b, no significant differences were observed among the contact angles of different samples. The highest contact angle, 158.2°, was recorded for D3LTE-20, while the lowest, 152.3°, was observed for D3LTE-15. Although samples fabricated with different printing parameters exhibit variations in pore size and microscale surface roughness, no clear correlation was found between these parameters and the resulting contact angle. This is because the sample surface consists primarily of PLA, with only minor differences in microscale roughness. The improvement in hydrophobicity is predominantly attributed to the low surface energy provided by the modification process and the introduction of nanoscale roughness [34]. Consequently, all modified samples achieved superhydrophobicity, and the variation in contact angles did not follow a distinct pattern.

**Figure 7:**
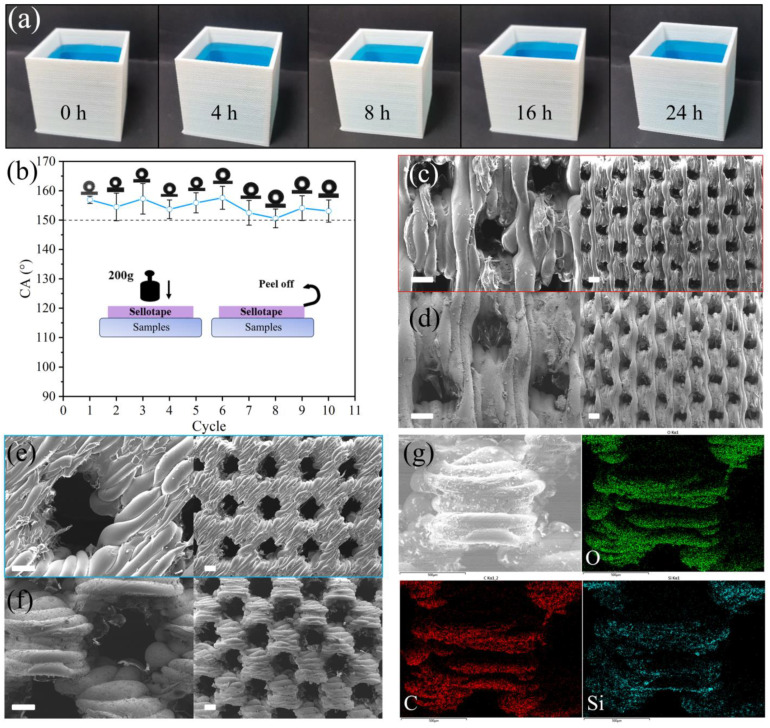
(**a**) Experiment demonstrating the internal water pressure resistance of modified D3LTE over 24 h. (**b**) Contact angle variation of modified D3LTE during the peeling test. SEM images of (**c**) the sample with a 0.4 mm pore size and (**d**) its modified version. SEM images of (**e**) unmodified D3LTE and (**f**) modified D3LTE. (**g**) Elemental mapping images of modified D3LTE. Scale bar is 200 µm.

Figure 7a illustrates the ability of the sample surface to withstand continuous water pressure. Over a 24 h period, no water leakage was observed, demonstrating the material’s excellent resistance to liquid infiltration. During the experiment, a notable phenomenon was observed: the water inside the sample evaporated rapidly and had to be continuously replenished to maintain the liquid level. This can be attributed to the high surface roughness and porosity of the modified structure based on Cassie–Baxter theory [33,35], which enhances the evaporation rate by increasing the water–air interface area and reducing the contact between water and the underlying solid, thus minimizing heat dissipation. As shown in Figure 7b, the sample surface was repeatedly subjected to peeling tests. After ten cycles, the modified D3LTE retained its hydrophobicity, with a contact angle of 153.1°, demonstrating strong adhesion stability. This stability is attributed to the excellent mechanical properties of PDMS, which serves as an effective binder between silica nanoparticles and the porous skeleton, preventing detachment under mechanical stress [36].

To confirm the presence of silica nanoparticles on the sample surface, SEM images of unmodified and modified D3LTE are shown in Figure 7e and Figure 7f, respectively. The deposition of nanoparticles on the surface increases the actual surface area, thereby enhancing the surface roughness (defined in the Wenzel model [33] as the ratio of actual to apparent surface area). This increased roughness contributes to the improved hydrophobicity observed after modification. Further elemental analysis was conducted using EDS, as shown in Figure 7g. Three characteristic elements—C, O, and Si—were detected on the modified surface. Si is uniformly distributed, confirming the successful incorporation of silica nanoparticles. Additionally, Figure 7c,d display SEM images of samples with a pore size of 0.4 mm, revealing similar roughness variations and further supporting the uniformity and effectiveness of the surface modification process.

### 3.3. Oil–Water Separation Experiment

**Figure 8:**
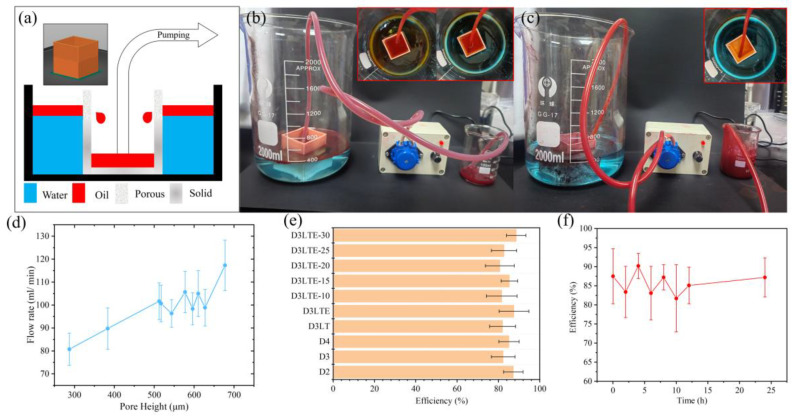
(**a**) Schematic diagram of the continuous pumping oil–water separation experiment. (**b**) Photographs taken during the continuous pumping oil–water separation process. (**c**) Image of the separated oil and water after the experiment. (**d**) Flow rate of different samples (based on pore height) during oil–water separation. (**e**) Separation efficiency of different samples. (**f**) Efficiency variation of modified D3LTE during continuous oil–water separation over 24 h.

To achieve continuous oil–water separation, an experimental setup was designed under laboratory conditions, as illustrated in Figure 8a. Modifications were made to the sample model to address the issue of secondary contamination. Since oil inherently passes through porous structures via capillary action, leading to potential leakage, the lower half of the sample was designed as a solid, non-porous region, providing an integrated oil storage capability. As shown in Figure 8b, the sample was placed in a beaker and secured underwater using double-sided tape. A controlled volume of water and cyclohexane was then introduced into the system. Due to the negative capillary effect induced by the superhydrophobic surface, water was effectively repelled, whereas cyclohexane permeated the porous structure under the combined effects of liquid pressure and capillary forces and was subsequently collected using an electric pump. After separation, as depicted in Figure 8c, the water surface remained clear and free of oil contamination, demonstrating effective oil removal.

Figure 8d presents the flow rate of different samples during oil–water separation. The flow behavior of cyclohexane through the porous structure can be analyzed using Darcy’s law [37], which describes the relationship between flow velocity and permeability in a porous medium:

$$v=\frac{k\;\Delta P}{\mu \;L}$$

where *v* is the fluid velocity, *k* is the permeability of the porous medium, *μ* is the dynamic viscosity of cyclohexane, ∆*P* is the pressure difference across the porous structure, and *L* is the thickness of the porous layer. Since permeability *k* is proportional to the square of the pore radius (k∝ r^2^), Darcy’s law predicts that larger pores should lead to significantly higher flow rates. This theoretical trend aligns generally with our experimental results, where an increasing trend in flow rate is observed with the enlargement of pore size (height). D3LTE, with a pore height of 677.3 µm, exhibits the highest flow rate (117.3 mL/min), while D3LTE-30, with a smaller pore height of 287.7 µm, demonstrates the lowest flow rate (80.7 mL/min). These results confirm that pore size plays a dominant role in controlling flow rate, as larger pores reduce flow resistance and enhance permeability. However, the observed flow rate differences between samples are somewhat smaller than those predicted by Darcy’s law. According to theoretical calculations, the flow rate should be scaled with r^2^, suggesting that the sample with a larger pore size should exhibit a significantly higher flow rate than what is experimentally recorded. This deviation may be attributed to additional factors beyond permeability. One key factor to consider in low-pressure filtration environments is the capillary effect [29], which becomes more significant in smaller pores. The Laplace equation shows that smaller pores generate higher capillary pressure, which increases the resistance to fluid entry and may suppress flow despite higher theoretical permeability. In this experiment, the small-pore sample (D3LTE-30) exhibits greater capillary resistance, counteracting the permeability-driven flow enhancement predicted by Darcy’s law. This effectively narrows the flow rate difference between samples with large and small pores, explaining why the experimental results deviate from purely permeability-based predictions. Therefore, the observed non-linearity in the flow response should not be interpreted as a violation of Darcy’s law but rather as a reflection of complex interfacial phenomena, particularly in porous membranes with superhydrophobic modifications.

Figure 8e presents the separation efficiency of different samples, with D3LTE-30 exhibiting the highest efficiency at 88.6%. As observed in Figure 8c, residual cyclohexane remains on the sample surface and water surface after separation, which accounts for the inability to achieve 100% separation efficiency. Additionally, a 24 h continuous oil–water separation test was conducted using D3LTE. After 24 h, the separation efficiency remained at 87.2%, demonstrating the sample’s potential for long-term oil–water separation applications.

The above analysis provides valuable insights into the practical application of the samples. However, these results are based on preliminary exploratory experiments conducted under controlled laboratory conditions. In real-world applications, the fluid environment is significantly more complex and dynamic. Therefore, further studies are required to validate these findings and optimize the samples for practical implementation.

**Figure 9:**
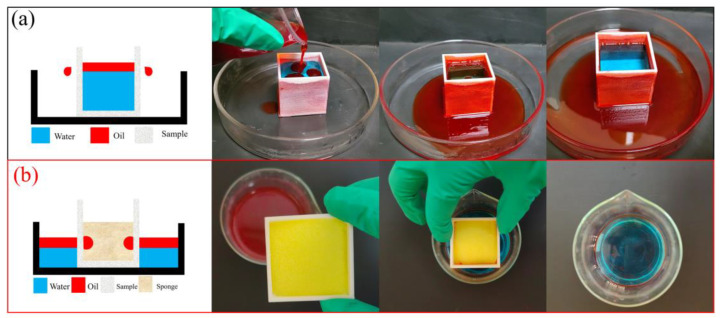
(**a**) Schematic diagram of the internal oil–water separation method and its corresponding photos. (**b**) Schematic diagram of the adsorption-based oil–water separation method combined with the sponge and its corresponding photos.

Due to the superhydrophobic porous structure of the sample, alternative oil–water separation methods can be employed. As illustrated in Figure 9a, cyclohexane and water are introduced into the internal space of the sample, where cyclohexane selectively permeates through the porous structure, enabling separation. Additionally, the sample can be combined with a sponge to achieve an adsorption-based oil–water separation process (Figure 9b). When a sponge is placed inside the sample, the cyclohexane that passes through the porous surface is immediately absorbed by the sponge, enabling the removal of cyclohexane from the water.

## 4. Conclusions

In this study, a 3D-printed superhydrophobic porous architecture was successfully fabricated using FFF technology, demonstrating efficient and continuous oil–water separation. By optimizing printing parameters, including extrusion multiplier, printing speed, and layer height, a diamond pore structure was developed, minimizing overhang defects while achieving uniform pores with a maximum deviation of 36.1 µm.

Although the polymer matrix (PLA) itself is not chemically modified in this study, its mechanical and thermal properties provide a robust platform for structure-controlled separation membranes. Future studies will consider polymer blending or copolymerization to further tune surface or bulk properties.

Superhydrophobic modification was achieved via spray-coating with silica nanoparticles and PDMS, increasing the water contact angle of D3LTE from 79.2° to 158.2°. The coated structures exhibited strong mechanical stability, retaining a contact angle of 153.1° after 10 peeling cycles and withstanding internal water pressure for 24 h without leakage.

Oil–water separation experiments demonstrated significant performance advantages. In a continuous pumping system, the material (D3LTE) maintained 87.2% separation efficiency over 24 h, with the highest efficiency recorded at 88.6% (D3LTE-30). Flow rate analysis confirmed the strong influence of pore size, with D3LTE (677.3 µm pores) achieving a flow rate of 117.3 mL/min, while D3LTE-30 (287.7 µm pores) exhibited a reduced flow rate of 80.7 mL/min. These findings establish a new pathway for scalable, energy-efficient oil–water separation, offering a cost-effective and industrially viable solution for environmental remediation and wastewater treatment.

Looking forward, several directions remain to be explored to further advance the applicability of this approach. These include the use of large-scale industrial 3D printers to fabricate porous structures while maintaining high geometric fidelity, the long-term environmental assessment of potential nanoparticle release during continuous operation, and structural optimization for effective separation under complex real-world conditions, such as viscous oils or saline wastewater. These future efforts will be essential for translating the current laboratory-scale success into robust, field-deployable solutions.

## Figures and Tables

**Table 1:** Printing parameters for the diamond porous structure.

Sample	t (mm)	Speed (mm s^−1^)	T (°C)	Extrusion Multiplier	Layer Height (mm)
D1	0.8	30	215	1	0.05
D2	1.6	30	215	1	0.05
D3	2.0	30	215	1	0.05
D4	2.4	30	215	1	0.05
D3LT	2.0	30	205	1	0.05
D3LTF	2.0	45	205	1	0.05
D3LTE	2.0	30	205	0.9	0.05
D3LTE-10	2.0	30	205	0.9	0.10
D3LTE-15	2.0	30	205	0.9	0.15
D3LTE-20	2.0	30	205	0.9	0.20
D3LTE-25	2.0	30	205	0.9	0.25
D3LTE-30	2.0	30	205	0.9	0.30

## Data Availability

The original contributions presented in this study are included in the article/Supplementary Material. Further inquiries can be directed to the corresponding author.

## Supplementary Materials

The following supporting information can be downloaded at: https://www.mdpi.com/article/10.3390/polym17111465/s1, Figure S1. (a) Three-view schematic diagram of the square pore structure; (b) Detailed top-view illustration; (c) Complete 3D model of the sample; (d) Magnified detail of the pore structure; (e) Digital model of the sliced sample prepared for 3D printing. SEM images of printed square pore structures (f) S1, (g) S2, (h) S3 and (i) S4. (Scale bar is 200 µm); Table S1. Printing parameter for the diamond porous structure; Figure S2. (a) square and (b) diamond pore structure and printing details.
