# Supplementary material for: Innovative 3D-Printed Superhydrophobic Porous Architectures for Continuous Oil–Water Separation
Source: Polymers (Basel). 2025 May 25;17(11):1465. doi: 10.3390/polym17111465 (PMC12157158; doi:10.3390/polym17111465)
Supplement: Supplementary file 1 [file polymers-17-01465-s001.zip › polymers-3655703-supplementary.pdf]

Supporting Information

Innovative 3D-Printed Superhydrophobic Porous Architectures for Continuous Oil-Water Separation

Xiaolong Wang[1,2], Jingjing An[2], Alaa Hassan[1], Qingsen Gao[3], Xianhu Liu[2,*]and Hakim Boudaoud[1]

[1] Équipe de Recherche sur les Processus Innovatifs (ERPI), Université de Lorraine, F-54000 Nancy, France; xiaolong.wang@univ-lorraine.fr (X.W.); alaa.hassan@univ-lorraine.fr (A.H.); hakim.boudaoud@univ-lorraine.fr(H.B.)
[2] College of Materials Science and Engineering, Zhengzhou University, Zhengzhou 450001, China; xianhu.liu@zzu.edu.cn (X.L.)
[3] Institute of Polymer Materials, Friedrich-Alexander-University Erlangen-Nuremberg, Martensstr. 7, 91058, Erlangen, Germany; qingsen.gao@fau.de (Q.G.)
* Correspondence: xianhu.liu@zzu.edu.cn

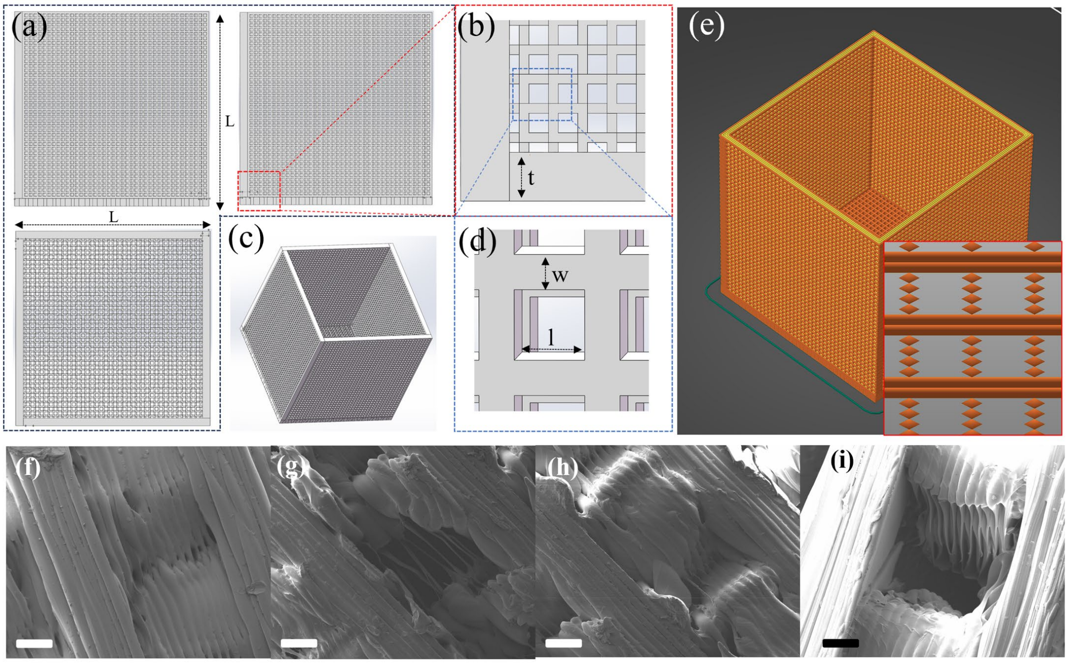

Figure S1. (**a**) Three-view schematic diagram of the square pore structure; (**b**) Detailed top-view illustration; (**c**) Complete 3D model of the sample; (**d**) Magnified detail of the pore structure; (**e**) Digital model of the sliced sample prepared for 3D printing. SEM images of printed square pore structures (**f**) S1, (**g**) S2, (**h**) S3 and (**i**) S4. (Scale bar is 200 μm).

Table S1. Printing parameter for the diamond porous structure.

**Sample**	t(mm)	Speed (mm s^{-1})	T (°C)	Extrusion Multiplier	Layer height(mm)
**S1**	2	30	215	1	0.05
**S2**	2	30	215	0.9	0.05
**S3**	2	30	205	1	0.05
**S4**	2	30	205	0.9	0.05

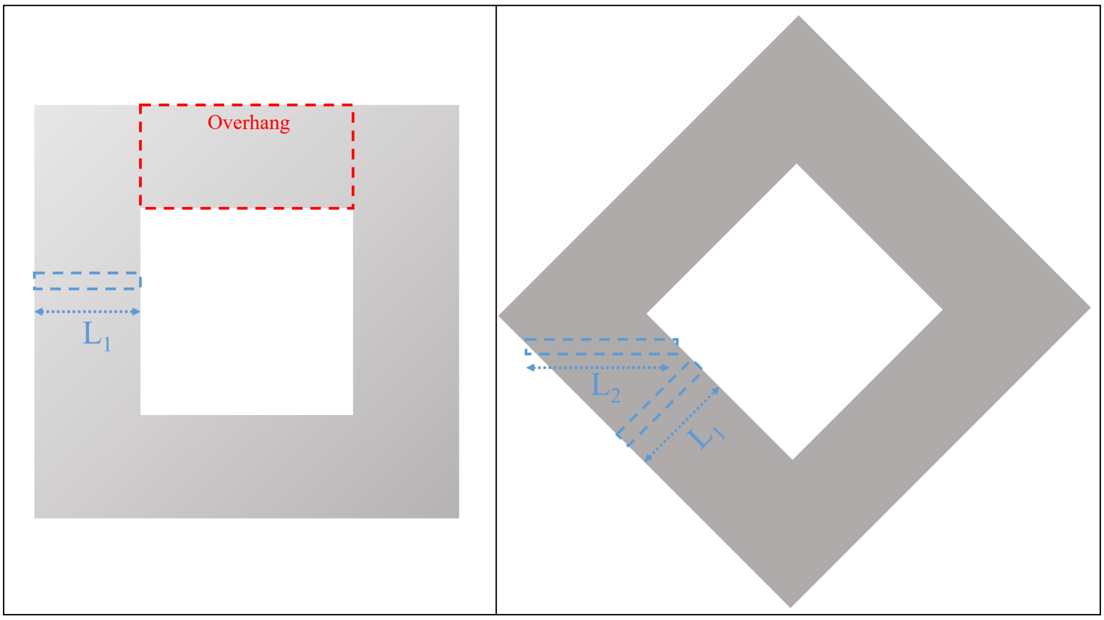

Figure S2. (**a**) square and (**b**) diamond pore structure and printing details.
